# Assessing the risk of cardiovascular diseases in relation to shisha smoking among adults in Qatar: An analytical cross-sectional study

**DOI:** 10.18332/tid/156678

**Published:** 2023-02-09

**Authors:** Elhassan Mahmoud, Ahmed Eliwa, Yasmin Elsalakawi, Alghalya Al-emadi, Fathima Mahmood, Noof Al-Qahtani, Wafaa Al-Mannai, Habib H. Farooqui, Susu M. Zughaier

**Affiliations:** 1 Basic Medical Sciences Department, College of Medicine, QU Health, Qatar University, Doha, Qatar; 2 Population Medicine Department, College of Medicine, QU Health, Qatar University, Doha, Qatar

**Keywords:** cardiovascular diseases risk, heart attack, shisha, waterpipe, hookah

## Abstract

**INTRODUCTION:**

Tobacco smoking is a preventable cause of disease and death worldwide. Shisha has become a popular method of smoking tobacco. In Qatar, the prevalence of smoking in 2019 was 25.2%, of which 20.9% was smoking shisha. Shisha smoking is thought to have a harmful effect on the cardiovascular system. The main objective of this study was to understand the relationship between shisha smoking and cardiovascular disease risks.

**METHODS:**

All data were obtained from the Qatar Biobank (QBB). The study population consisted of 1045 individuals, which included cases defined as participants who had a history of angina, heart attack and/or stroke and their matched healthy controls for age and gender. The measurement of both the exposure and the outcome was done through the survey provided by QBB. A conditional logistic regression model was used to assess the association between smoking and cardiovascular disease (CVD), and adjusted odds ratio (AOR) and 95% confidence intervals (CI) were calculated after adjusting for covariates.

**RESULTS:**

After adjusting for hypertension diagnosis, diabetes diagnosis, dyslipidemia diagnosis, abdominal obesity, and sedentary lifestyle, exclusive shisha smokers had 1.65 times higher odds of reporting cardiovascular disease diagnoses compared to non-smokers (95% CI: 0.71–1.91). Dual shisha and cigarette smokers also had 1.47 times higher odds of reporting cardiovascular disease diagnoses compared to non-smokers (95% CI: 0.88–2.45). CVD cases had a younger median age of initial shisha smoking compared to controls (20 years vs 25 years, p=0.003).

**CONCLUSIONS:**

Shisha smoking was associated with an increased risk of developing cardiovascular disease. However, this association did not reach the level of statistical significance within this study. A finding to consider that showed strong evidence is the younger age of initial shisha smoking in cases. Further studies are needed to demonstrate the true relationship between shisha smoking and cardiovascular disease.

## INTRODUCTION

Cardiovascular diseases (CVDs) are the major cause of death worldwide. In 2019, an estimated 17.9 million individuals died from CVDs, accounting for 32% of all global deaths^1^. Eighty-five per cent of these deaths were caused by a heart attack or a stroke^1^. More than three-quarters of CVD deaths occur in low- and middle-income nations^1^. According to Qatar’s planning and statistical authorities, angina and stroke were among the leading causes of mortality in 2017, accounting for 32% of the deaths in Qatar^2^. The inter-heart study of myocardial infarction cases in 52 countries, identified nine modifiable factors that account for myocardial infarction in both genders^3^. These factors include smoking, hypertension, diabetes, abdominal obesity, psychosocial factors, fruit, vegetable, and alcohol consumption, and regular physical activity^3-6^.

Tobacco smoking is a preventable cause of disease and mortality worldwide^7^. Shisha has become lately a popular method of smoking tobacco^7^. Shisha is sometimes referred to as waterpipe, hookah, and narghile. The global percentage of shisha smokers is continuously increasing^7^. Eastern Mediterranean and European nations have the highest rates of shisha smoking, which appears to be more common among teenagers than adults^8^. In Qatar, many individuals smoke cigarettes or traditional hookahs^9^. A study done in 2019 showed that 25.2% (n=6904) of a sample in Qatar smoke tobacco^10^; of the 6904, 8.3% (n=570) were shisha smokers^11^.

Shisha smoking has a harmful effect on the body and cardiovascular system^12^. Shisha, like cigarettes, burns tobacco which produces toxic compounds including nitrosamine and polycyclic aromatic hydrocarbons, which are both carcinogens and increase the risk of developing CVD^13,14^. Oxidative stress is believed to be a major contributor to the pathophysiology of shisha smoking, which is associated with an increased risk of CVD and death with long-term use^14^. It has been shown that there is a positive association between shisha smoking and cancer risk, specifically lung and esophageal cancers^15^. Additionally, shisha smoking regularly is associated with respiratory complications, including reduced pulmonary functions and increased risk for COPD^16^. Many studies have found that shisha smokers’ systolic and diastolic blood pressure and heart rate increase after smoking^17^. In contrast to cigarette smoking, less is known regarding the health consequences of shisha use^7^. This study aims to assess the association between shisha smoking and CVD among the population of Qatar Biobank (QBB). This study is important as the association between shisha smoking and CVD has not been studied thoroughly, and hence, it is perceived in the population that shisha smoking is less harmful compared to cigarette smoking^18^.

## METHODS

### Study design and settings

The design of this study is analytical cross-sectional. Data were collected from QBB (https://www.qatarbiobank.org.qa/), which is a program that was started in December 2012 to collect biological samples and data on the health and lifestyle of a portion of Qatar’s population. Participants from QBB are aged ≥18 years and are either Qataris or long-term residents of Qatar (at least 15 years)^19^. Information obtained from QBB included demographics (age, gender, nationality, BMI, etc.), medical history (hypertension, diabetes, medications, etc.), laboratory measurements (cholesterol, LDL, glucose, insulin, etc.), and smoking status (shisha smoker, cigarette smoker, previous smoker, never smoker) for all participants.

### Definition of cases and controls

The study population consisted of 1045 individuals from the QBB cohort who had reported diagnoses of CVD and their matched healthy controls. The measurement of both the exposure and the outcome was done through the survey provided by QBB. A case was defined as a participant who reported a history of heart attack, angina and/or stroke diagnosis. This was done through QBB’s survey which asked the question: ‘Has a doctor ever told you that you have or had any of the following conditions?’. Inclusion criterion was those who met the case definition. Those who did not meet QBB’s eligibility criteria were excluded. A control was defined as a patient who did not report a history of heart attack, angina and/or stroke. QBB had a total of 209 participants with CVD in their database, and all of them were included in our study. Each case was matched to four controls for age and gender randomly (1:4). The total study population was 1045 after matching for age (±1 year) and gender. For instance, a male patient aged 45 years who reported a history of CVD diagnosis was matched with 4 male controls who were aged 44–46 years. This was done to increase the power of the study.

### Smoking status

Smoking history was determined from the survey. The survey asked: ‘Do you currently smoke?’. Participants were then categorized as current or former smokers, accordingly. The survey also asked questions such as ‘what do/did you usually smoke?’ and participants were categorized as cigarette or shisha smokers, accordingly. Participants who only smoked shisha were categorized as exclusive shisha smokers. Participants who only smoked cigarettes were categorized as exclusive cigarette smokers. Participants who smoked both cigarettes and shisha were categorized as dual shisha and cigarette smokers. The initial age of shisha smoking was also determined from the survey, which asked the question: ‘How old were you when you first started smoking waterpipe (shisha)?’.

### Other variables

Sedentary lifestyle was determined if the participants’ level of activity in their main occupations involved them sitting most of the time. Participants were considered obese if their BMI was ≥30 kg/m^2^. Abdominal obesity was defined as a waist-to-hip ratio >0.85 for females and >0.9 for males. Low income was defined as a monthly salary of ≤20000 QAR (100 Qatari Riyals about US$27). Medical diagnoses and medications were self-reported by participants. For example, a participant was considered to have history of diabetes diagnosis if he/she answered ‘Yes’ to the question: ‘Has a doctor ever told you that you had or have diabetes?’. Laboratory biochemical values, such as total cholesterol, were measured by QBB at the time of enrollment of volunteers. Monocyte to HDL ratio was calculated by dividing monocyte percentage by HDL cholesterol levels^20^. HOMA2 IR levels were calculated using University of Oxford’s HOMA calculator. Participants were considered to be fasting if their fasting time was ≥8 hours. Missing data in the study range from 14 to 125 participants. Smoking history had 94 missing responses, of which 76 were controls (9.1%) and 19 were cases (8.6%).

### Statistical analysis

Mean and standard deviation were used to express normally distributed continuous variables, and median and interquartile range (IQR) were used to express continuous variables that are not normally distributed. Frequency and percentage were used to express categorical variables.

The chi-squared test was used to compare categorical variables between cases and their matched controls. The independent t-test was used to compare the means of normally distributed continuous variables, and the Wilcoxon rank-sum test was used to compare the medians of continuous variables that are not normally distributed. A conditional logistic regression model was used to assess the association between smoking and CVD after adjusting for covariates. Confounding variables were determined after conducting a literature review on the variables of interest and creating a corresponding Directed Acyclic Graph (DAG). Stata 17 was used to carry out all the statistical analyses, and exact p-values are reported.

### Ethics statement

The study was approved by QBB’s Institutional Review Board and researchers signed a non-disclosure agreement to maintain confidentiality before receiving the data from QBB.

## RESULTS

A total of 1045 participants were divided into two groups, controls (n=836) and cases (n=209), matched for age and gender. Table 1 shows the baseline characteristics of both groups including demographics, medical history, and smoking history. The average age of both groups was 55.6 years. There were no differences in height, weight, obesity, abdominal obesity and education level between the two groups. There was no difference in the systolic blood pressure (mmHg), while the diastolic blood pressure showed a difference with a mean of 70.4 mmHg in the control group compared with a mean of 68.6 in the cases (p=0.031). More cases reported sedentary lifestyle compared to controls (p=0.002). There was a difference in monthly income between the two groups, as cases had a higher proportion of people with low income (p=0.032). Cases were more likely to have diabetes, hypertension and dyslipidemia (p<0.001) and they were also more likely to be treated for these conditions (p<0.001).

**Table 1:** Baseline characteristics of cases with heart attack, angina, and/or stroke and matched controls for age and gender, Qatar, 2022 (N=1045)

*Characteristics*	*Controls (n=836) n (%)*	*Cases (n=209) n (%)*	*p*
**Demographics**			
**Age** (years), mean ± SD	55.6 ± 12.0	55.6 ± 12.0	1.00^a^
Gender (male)	552 (66.0)	138 (66.0)	1.00^b^
Nationality (Qatari)	666 (79.7)	163 (78.0)	0.59^b^
Height (cm), mean ± SD	181.9 ± 88.8	175.7 ± 85.6	0.36^a^
Weight (kg), mean ± SD	83.5 ± 16.0	83.4 ± 16.3	0.89^a^
Obesity*	411 (49.7)	107 (51.7)	0.61^b^
Abdominal obesity*	526 (63.8)	143 (69.1)	0.16^b^
SDP (mmHg), mean ± SD	125.5 ± 17.1	126.4 ± 17.2	0.51^a^
DPB (mmHg), mean ± SD	70.4 ± 10.7	68.6 ± 11.5	0.031^a^
Sedentary lifestyle*	322 (41.7)	107 (54.0)	0.002^b^
Education level (university or higher)*	414 (50.5)	89 (54.0)	0.052^b^
Low income*	354 (48.5)	109 (57.4)	0.029^b^
**Medical history**			
Diabetes diagnosis	361 (43.2)	132 (63.2)	<0.001^b^
Hypertension diagnosis*	259 (31.9)	104 (50.2)	<0.001^b^
Dyslipidemia diagnosis	403 (48.2)	143 (68.4)	<0.001^b^
Diabetes medication	260 (31.1)	109 (52.2)	<0.001^b^
Hypertension medication	213 (25.5)	91 (43.5)	<0.001^b^
Dyslipidemia medication	251 (30.0)	99 (47.4)	<0.001^b^
**Smoking history**			
Current smokers*	143 (18.8)	35 (18.3)	0.88^b^
Former smokers*	141 (18.6)	48 (25.1)	0.042^b^
**Smoking type**			
Exclusive shisha smokers*	53 (7.0)	13 (6.8)	0.94^b^
Exclusive cigarette smokers*	88 (11.6)	28 (14.7)	0.24^b^
Dual shisha and cigarette smokers*	143 (18.8)	42 (22.0)	0.32^b^

SDP: systolic blood pressure. DPB: diastolic blood pressure. IQR: interquartile range. SD: standard deviation.

a Independent t-test.

b Chi-squared.

c Wilcoxon rank-sum.

* Missing participant responses range from 14 to 125.

**Figure 1:**
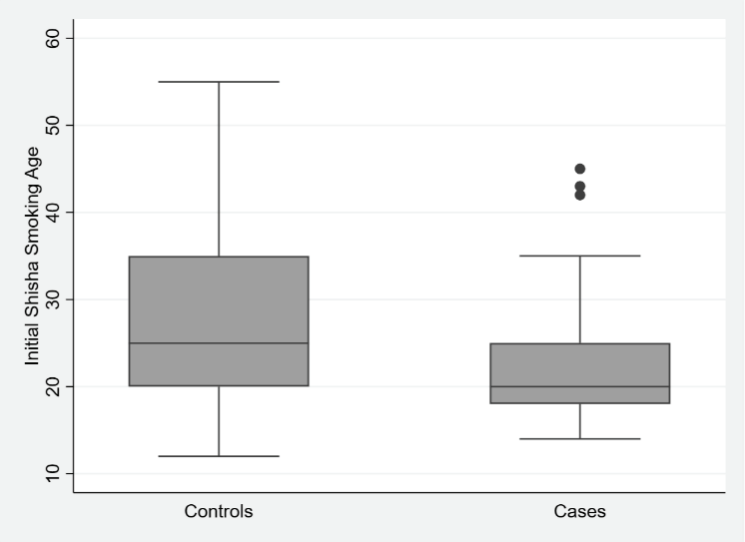
Box plot of initial shisha smoking age by heart attack, angina, and/or stroke history (p=0.003), Qatar, 2022

In this study, smoking history was positive in 284 of the participants. Current smokers accounted for 18.3% of the cases and 18.8% of the controls (p=0.88). The percentage of former smokers was higher in cases compared to controls (25.1% vs 18.6%, p=0.042). Exclusive shisha smokers constituted 6.8% of the cases and 7% of the controls (p=0.94). Exclusive cigarette smokers made up 14.7% of the cases and 11.6% of the controls (p=0.24); 22% of cases and 18.8% of controls were dual shisha and cigarette smokers (p=0.32). The median age of initial shisha smoking (Figure 1) was lower in cases compared to controls (20 years vs 25 years, p=0.003).

Table 2 depicts the measured laboratory biochemical values of cases and controls. The data revealed that cases have higher levels of HbA1c compared to controls (p<0.001). The cases showed better lipid profile compared with controls. The mean of total cholesterol is lower among cases with CVD compared to controls (4.7 vs 5.0 mmol/L, p=0.003). LDL cholesterol levels were lower in cases compared with controls (2.7 vs 3.0 mmol/L, p=0.002). HDL cholesterol levels are different between cases and controls (p=0.042) with means of 1.2 and 1.3 mmol/L, respectively. For renal function tests, serum creatinine was higher in the cases with a median of 74.0 µmol/L, while the control group had a median of 72.0 µmol/L (p=0.019). The mean of urea was 5.8 mmol/L in the cases compared with 4.9 mmol/L in the controls (p<0.001). The levels of homocysteine differ between the cases and controls with medians of 11.0 and 10.0 µmol/L, respectively (p=0.034).

**Table 2:** Selected laboratory variables for cases with heart attack, angina, and/or stroke and matched controls for age and gender, Qatar, 2022 (N=1045)

*Variables*	*Controls (n=836) Mean ± SD or Median (IQR)*	*Cases (n=209) Mean ± SD or Median (IQR)*	*p*
Fasting glucose (mmol/L)	5.6 (5.0–7.4)	6.1 (5.2–7.8)	0.018^c^
Fasting C-Peptide (ng/mL)	2.4 ± 1.1	2.5 ± 1.5	0.48^a^
Fasting insulin (µU/mL)	11.2 (7.7–16.9)	11.0 (8.0–16.1)	0.85^c^
HbA1c (%)	5.8 (5.4–6.9)	6.5 (5.6–8.2)	<0.001^c^
Total cholesterol (mmol/L)	5.0 ± 1.1	4.7 ± 1.1	0.003^a^
HDL cholesterol (mmol/L)	1.3 ± 0.4	1.2 ± 0.4	0.042^a^
LDL cholesterol (mmol/L)	3.0 ± 1.0	2.7 ± 1.1	0.002^a^
Triglyceride (mmol/L)	1.3 (1.0–1.8)	1.5 (1.0–2.1)	0.026^a^
Creatinine (µmol/L)	72.0 (61.0–82.0)	74.0 (63.0–87.0)	0.019^c^
Monocyte to HDL ratio	6.6 ± 2.6	6.7 ± 2.4	0.60^c^
Urea (mmol/L)	4.9 ± 1.6	5.8 ± 3.6	<0.001^a^
Ferritin (µg/L)	78.2 (34.0–140.5)	69.0 (28.2–122.0)	0.086^a^
Homocysteine (µmol/L)	10.0 (8.0–12.5)	11.0 (8.4–13.0)	0.034^c^
Total dihydroxyvitamin D (ng/mL)	20.0 (14.0–28.0)	21.0 (15.0–29.0)	0.23^c^
HOMA2 IR	5.67 ± 2.67	6.06 ± 3.36	0.17^c^

HDL: high-density lipoprotein. LDL: low-density lipoprotein.

a Independent t-test.

b Chi-squared.

c Wilcoxon rank-sum.

IQR: interquartile range. SD: standard deviation.

Table 3 shows the conditional logistic regression model (matched for age and gender) analysis of the smoking effect, including exclusive shisha, exclusive cigarette, and dual shisha and cigarette smoking, on CVD diagnosis. After adjusting for covariates, including hypertension, diabetes, dyslipidemia, abdominal obesity, and sedentary lifestyle, exclusive shisha smokers had 65% higher odds of reporting cardiovascular disease diagnoses compared to non-smokers (95% CI: 0.78–3.48). Exclusive cigarette smokers had 39% higher odds of reporting cardiovascular disease diagnoses compared to non-smokers (95% CI: 0.79–2.43). Those who smoked both shisha and cigarettes had 47% higher odds of reporting cardiovascular disease diagnoses compared to non-smokers (95% CI: 0.88–2.45). However, these findings were not statistically significant at the given sample size. An analysis was conducted to explore the association between smoking and stroke and coronary artery disease separately (Supplementary file Tables S1 and S2).

**Table 3:** Association between smoking history and history of heart attack, stroke, and/or angina by multivariable conditional logistic regression, Qatar, 2022 (N=824)

*Variable*	*OR (95% CI)*	*p*	*AOR (95% CI)*	*p*
Exclusive shisha smokers	1.25 (0.63–2.49)	0.52	1.65 (0.78–3.48)	0.19
Exclusive cigarette smokers	1.55 (0.92–2.62)	0.10	1.39 (0.79–2.43)	0.25
Dual shisha and cigarette smokers	1.44 (0.90–2.30)	0.13	1.47 (0.88–2.45)	0.14

AOR: adjusted odds ratio; adjusted for hypertension diagnosis, diabetes diagnosis, dyslipidemia diagnosis, abdominal obesity, and sedentary lifestyle. In all, 84 participants were omitted because of all positive or all negative outcomes.

## DISCUSSION

This study focused on smoking, particularly shisha smoking, and the risk of developing CVD in adults residing in Qatar. This was done by analyzing the odds of CVD diagnosis after adjusting for covariates (hypertension, diabetes, dyslipidemia, sedentary lifestyle, and abdominal obesity). We found that exclusive shisha smokers had higher odds of reporting CVD diagnoses compared to non-smokers. However, there was little evidence against the null hypothesis at the given sample size. This may be attributed to the small sample size in which there was a very limited number of exclusive shisha smokers in the study (n=66), which potentially led to random error.

It was noted in the results that cases had a younger age of initial shisha smoking. This is probably because younger starting age will lead to a longer duration of exposure. However, the duration of shisha smoking was not assessed in this study because a large number of participants had missing information in this aspect. From a preventative perspective, doctors may consider paying more attention to raising younger adults’ awareness, which may help to reduce the duration of exposure.

No studies were conducted exclusively in Qatar on the association between CVD and shisha smoking. One study reported the prevalence of shisha and cigarette smoking in Qatar^11^. A recent study conducted in both Qatar and Lebanon showed that shisha smoking was associated with the presence of coronary artery calcium (CAC), which is a marker of coronary artery disease^21^. Another study showed that exclusive waterpipe smoking was linked to increased arterial stiffness in a community-based sample, which is a predictor of cardiovascular disease^22^. Several studies in the Middle East and South-East Asia have found an association between long-term waterpipe use and increased CVD risk, severity, and mortality^23-25^. Nonetheless, the majority of these studies have various limitations^26^. A study conducted in Saudi Arabia showed a significant association between shisha smoking and myocardial infarction^27^. However, the study conducted was not generalizable given its small sample size; in addition to having a low internal validity given the fact that the patients self-reported their diagnoses.

Those participants with a prior diagnosis of CVD had lower total cholesterol and LDL cholesterol levels compared to controls. This may be attributed to the fact that a larger proportion of the cases have a diagnosis of dyslipidemia, and are thus taking lipid-lowering agents. Furthermore, these patients were most likely prescribed statins as they are indicated for CVD patients^28^. This is consistent with the results of a prior study that showed that patients with myocardial infarction or angina had improved lipid profiles and a lower prevalence of dyslipidemia one year after percutaneous coronary intervention (PCI)^29^.

### Strengths and limitations

One of the major strengths of this study is that cases and controls were matched for age and gender, which are both confounders. In addition, we adjusted for other covariates, including hypertension, diabetes, dyslipidemia, abdominal obesity, and sedentary lifestyle to eliminate the possibility that the variation in results between the two groups is due to differences in these variables. Another strength is that this is the first study conducted exclusively in Qatar that has ever been done to assess the effect of shisha smoking on the development of CVDs.

The study had some limitations. Among the cases (n=209), there were few exclusive shisha smokers (n=13) as most shisha smokers were cigarette smokers too. In addition, because of the nature of data collection and study design, a causal association could not be assessed. Moreover, QBB’s population is not representative of the general population of Qatar, which may limit the generalizability of our study.

There are some limitations regarding the outcome definition, as it is self-reported with no specific diagnostic criteria and missing data on the time of diagnosis. This study also did not assess other forms of tobacco smoking such as cigars. The frequency of exposure to shisha smoking was also not assessed, which warrants further investigations. To evaluate the association between CVD and shisha smoking and reduce the chance of error, more studies with larger sample sizes and proper designs are needed. For example, a prospective study or a study where directionality can be assessed.

## CONCLUSIONS

This was the first study conducted exclusively in Qatar to investigate the association between shisha smoking and the development of CVD. Shisha smoking is associated with increased risk of developing CVD. However, this association did not reach the level of statistical significance within the study. A key finding in this study is that the younger age to start shisha smoking is significantly associated with increased risk of CVD. Finally, studies with appropriate sample size, design, and measurement of exposure and outcome are required to demonstrate a true relationship between shisha smoking and CVD.

## Data Availability

The data supporting this research cannot be made available for privacy or other reasons. The cohort data used in this current study were obtained from Qatar Biobank repository in Doha, Qatar [https://www.qatarbiobank.org.qa/] under a Material Transfer agreement, and thus are not publicly available. Upon reasonable request, S. Zughaier (szughaier@qu.edu.qa) will facilitate communication with the Qatar Biobank.
