# Supplementary material for: Assessing the risk of cardiovascular diseases in relation to shisha smoking among adults in Qatar: An analytical cross-sectional study
Source: Tob Induc Dis. 2023 Feb 9;21:21. doi: 10.18332/tid/156678 (PMC9909683; doi:10.18332/tid/156678)
Supplement: Supplementary file 1 [file TID-21-21-s1.pdf]

Table S1. Association between smoking history and history of stroke by multivariable conditional logistic regression. Qatar, 2022 (N = 158).

Variable	OR (unadjusted) (95% CI)[a]	*p*-value[a]	OR (adjusted) (95% CI)[b]	*p*-value[b]
Exclusive shisha smokers	0.79 (0.08 – 8.01)	0.845	2.22 (0.11 – 44.93)	0.602
Exclusive cigarette smokers	3.14 (0.94 – 10.49)	0.063	3.85 (1.00 – 14.76)	0.049
Dual shisha and cigarette smokers	2.61 (0.75 – 9.14)	0.132	4.20 (0.99 – 17.89)	0.052

OR: odds ratio, CI: confidence interval. 602 observations were omitted because of all positive or all negative outcomes. [a]: unadjusted OR and p-value, [b]: adjusted for hypertension diagnosis, diabetes diagnosis, dyslipidemia diagnosis, abdominal obesity, sedentary lifestyle.

Table S2. Association between smoking history and history of heart attack and/or angina by multivariable conditional logistic regression. Qatar, 2022 (N = 673).

Variable	OR (unadjusted) (95% CI)[a]	*p*-value[a]	OR (adjusted) (95% CI)[b]	*p*-value[b]
Exclusive shisha smokers	1.27 (0.62 – 2.61)	0.517	1.55 (0.71 – 3.40)	0.270
Exclusive cigarette smokers	1.30 (0.72 – 2.35)	0.378	1.14 (0.61 – 2.15)	0.675
Dual shisha and cigarette smokers	1.33 (0.80 – 2.22)	0.271	1.36 (0.79 – 2.36)	0.271

OR: odds ratio, CI: confidence interval. 200 observations were omitted because of all positive or all negative outcomes. [a]: unadjusted OR and p-value, [b]: adjusted for hypertension diagnosis, diabetes diagnosis, dyslipidemia diagnosis, abdominal obesity, sedentary lifestyle.

© 2023 Mahmoud E. et al.
